# Micro ecosystems from feed industry surfaces: a survival and biofilm study of *Salmonella *versus host resident flora strains

**DOI:** 10.1186/1746-6148-6-48

**Published:** 2010-11-02

**Authors:** Olivier Habimana, Trond Møretrø, Solveig Langsrud, Lene K Vestby, Live L Nesse, Even Heir

**Affiliations:** 1 Nofima Mat AS, Osloveien 1, N-1430 Ås, Norway; 2 National Veterinary Institute, Section of Bacteriology, P.O. Box 750 Sentrum, N-0106 Oslo, Norway

## Abstract

**Background:**

The presence of *Salmonella *enterica serovars in feed ingredients, products and processing facilities is a well recognized problem worldwide. In Norwegian feed factories, strict control measures are implemented to avoid establishment and spreading of *Salmonella *throughout the processing chain. There is limited knowledge on the presence and survival of the resident microflora in feed production plants. Information on interactions between *Salmonella *and other bacteria in feed production plants and how they affect survival and biofilm formation of *Salmonella *is also limited. The aim of this study was to identify resident microbiota found in feed production environments, and to compare the survival of resident flora strains and *Salmonella *to stress factors typically found in feed processing environments. Moreover, the role of dominant resident flora strains in the biofilm development of *Salmonella *was determined.

**Results:**

Surface microflora characterization from two feed productions plants, by means of 16 S rDNA sequencing, revealed a wide diversity of bacteria. Survival, disinfection and biofilm formation experiments were conducted on selected dominant resident flora strains and *Salmonella*. Results showed higher survival properties by resident flora isolates for desiccation, and disinfection compared to *Salmonella *isolates. Dual-species biofilms favored *Salmonella *growth compared to *Salmonella *in mono-species biofilms, with biovolume increases of 2.8-fold and 3.2-fold in the presence of *Staphylococcus *and *Pseudomonas*, respectively.

**Conclusions:**

These results offer an overview of the microflora composition found in feed industry processing environments, their survival under relevant stresses and their potential effect on biofilm formation in the presence of *Salmonella*. Eliminating the establishment of resident flora isolates in feed industry surfaces is therefore of interest for impeding conditions for *Salmonella *colonization and growth on feed industry surfaces. In-depth investigations are still needed to determine whether resident flora has a definite role in the persistence of *Salmonella *in feed processing environments.

## Background

*Salmonella *contaminations in animal feeds are important vectors for *Salmonella *infections in livestock farms [1-9]. The presence of *Salmonella enterica *serovars in feed ingredients is a well-known problem. Heat treatment of animal feed is central for ensuring microbial feed safety, despite the fact that some reports stated that thermal treatment conditions during pelleting are not effective for eliminating *Salmonella *contamination in feeds [10,11]. However, since most feed plants have good control over heat treatment procedures, feed contamination is most likely due to recontamination from feed processing environments [12]. Despite strict safety measures implemented by the feed industry in Norway, certain *Salmonella *serovars (e.g. Agona, Montevideo and Senftenberg) are routinely isolated, and molecular typing data indicate that specific strains have persisted in certain feed processing environments for years [6].

The choice of disinfectants and proper cleaning practices are critical factors for eliminating *Salmonella *[2], yet the use of cleaning and disinfection is limited in feed processing environments, as a means to ensure dry production environments. The overall dry production environment in the feed production industry limits bacterial growth, including growth of *Salmonella*. However, on certain production surfaces, condensation caused by temperature variations may provide sufficient moisture to bacterial niches, in which *Salmonella *and other bacteria are likely to grow and form biofilms [13]. In addition, lack of cleaning may lead to a build-up of organic material as well as settlement and establishment of other microorganisms on feed processing surfaces [13,14]. Recent studies have shown that biofilm formation plays a role in the ability for *Salmonella *to survive and persist in feed and food processing environments [14-16]. The presence of such resident flora on feed processing surfaces could potentially promote microbial adhesion and subsequent biofilm formation and persistence of unwanted pathogenic bacteria. Studies have shown that the presence and type of resident biofilms on a surface could significantly influence the initial adhesion of *Listeria monocytogenes *and *Escherichia coli *O157:H7 and their subsequent surface colonization [17,18]. Furthermore, recent studies have revealed that *E. coli *O157:H7 biofilm formation abilities were increased by the presence of some beef processing resident flora isolates [18,19].

The persistence and epidemiology of *Salmonella *in feed factories is relatively well documented [6,14,15,20,21]. However, other bacteria in feed factories have been less investigated. There is no knowledge as to whether the composition of a resident bacterial flora affects the growth and survival of *Salmonella *in feed processing environments. An improved understanding of the microbial ecology of feed production facilities and their potential interactions with *Salmonella *would be important in order to introduce improved strategies to eliminate *Salmonella *in the feed industry. In this study, we characterized resident aerobic bacterial flora at four critical control points of two feed production plants using 16 S rDNA sequencing of bacterial isolates. Survival properties under different environmental conditions and susceptibility to disinfectants were examined for selected isolates. Furthermore, the effects of selected resident flora isolates on the biofilm formation of *Salmonella *were investigated.

## Methods

### Bacterial strains and growth conditions

*Salmonella *strains used in this study are listed in Table 1. Four strains representative of the serovars of *Salmonella *spp. dominating in Norwegian feed factories were chosen, as well as the laboratory strain *Salmonella *Typhimurium ATCC 14028.

**Table 1:** Bacterial isolates used in this study

Bacterial strain	Origin/Reference
*S*. Agona 71-3^a, b^	FF; [6]
*S*. Agona 71-4^a^	FF; [14]
*S*. Montevideo 1900^a^	FF; [6]
*S*. Senftenberg 1702-1^a^	FF; [6]
*S*. Typhimurium ATCC 14028^a^	Chicken; control strain; ATCC
Corynebacterium sp.1^a^	FF; This study
Corynebacterium sp.2^a^	FF; This study
*Staphylococcus piscifermentans *1^a, b^	FF; This study
*Staphylococcus piscifermentans *2^a^	FF; This study
*Staphylococcus saprophyticus*^a^	FF; This study
*Pantoea agglomerans *1^a, b^	FF; This study
*Pantoea agglomerans *2^a^	FF; This study
*Pseudomonas *sp.1^a, b^	FF; This study
*Pseudomonas *sp.2^a^	FF; This study

^a ^Strains used for survival experiment

^b ^Strains selected for biofilm experiment

FF: Feed factory strain

The five *Salmonella *spp. strains were made rifampicin-resistant (Rif^R^) by sub culturing at 30°C in Tryptone Soy Broth (TSB; Oxoid, Hampshire, England) with increasing concentrations of rifampicin (Sigma-Aldrich, MO, USA), to 200 μg/mL. *Salmonella *isolates were cultivated at 30°C in TSB broth and Tryptone Soy Agar (TSA; Oxoid, Hampshire, England), when required, rifampicin was added to a final concentration of 100 μg/mL. No differences in growth rates were observed between Rif^R ^*Salmonella *strains and their *Salmonella *wild type counterparts. For microscopic observations the pGFP-uv plasmid (CLONTECH laboratories, Palo Alto, USA) was electroporated into *S*. Agona 71.3 competent cells as previously described [22]. Bacterial sampling in two factories producing fish feed (Plant A) and animal feed (Plant B) was performed at four routine sampling sites (two from the pre-heat-/raw ingredient region and two from the post-heat-/product region). Samples were obtained by combined scraping and surface swabbing of feed ingredients and feed contact surfaces. Swabs were added to 10 mL peptone water, mixed and 1 mL dilutions were then transferred to Petrifilm Aerobic plate counts (3 M, Skjetten, Norway). Total aerobic bacteria and lactic acid bacteria were isolated from Petrifilm Aerobic Count Plates and de Man, Rogosa, Sharpe agar (MRA), respectively, after 30°C incubation. For bacterial identification, each colony (up to *n *= 20) within a sector of the Petrifilm was isolated. The isolates were grown to pure culture in tryptone soya agar (TSA, Oxoid) at 30°C and stored in TSB with 15% glycerol at -80°C. Identification of isolates was performed by 16 S rRNA gene sequencing of approximately 500 bp PCR amplicons encompassing the V1 to V3 regions as previously described [23]. Sequence homologies were identified using Genbank BLAST [24]. Isolated resident bacteria used for survival and biofilm experiments are listed in table 1.

### Survival experiments

To obtain information on temperature and humidity conditions near sampling sites, logging devices (EL-USB-2, Lascar Electronics Ltd., Salisbury, UK) were placed in Plant A for automatic logging of temperatures and relative humidities (RH) for an approximate seven day production period. Based on recorded data, two temperatures and humidity conditions were selected for studies of bacterial survival on stainless steel surfaces. As a model for dry conditions, 30°C and 35% RH parameters were chosen, whereas the model used for humid conditions was fixed at 12°C and 85% RH. The experimental system used for studying bacterial survival was in accordance with the previous description [25]. Adjustment of RH levels was achieved by saturated potassium chloride and saturated potassium acetate solutions for 85% RH and 35% RH ambient conditions, respectively. Sealed boxes with 35% RH were placed in an incubator at 30°C for 1, 7, 14 and 28 days and boxes with 85% RH were placed at 12°C for the same time period. Sampling and determination of viable bacterial counts was performed as described [25] and the number of viable cells was determined by plating to TSA-agar. The experiment was conducted in triplicate.

### Disinfection tests

In all bactericidal tests, bacteria were exposed to the lowest recommended user-concentration of disinfectants in the presence of bovine serum albumin (BSA) (0.3% w/v) for 5 min at 20°C. The disinfection test used in this study was based on the European surface test (EN 13697), and was performed as previously described [13]. Three disinfectants were selected for bactericidal testing (Table 2). The selection was partially based on disinfectants representing the different categories of disinfectants used in Norwegian feed factories, and partially on these being among the most effective disinfectants against *Salmonella *dried on stainless steel [13]. Dey/Engley neutralizing broth (Difco) was used for diluting and neutralizing the disinfectants after exposure. The neutralization procedure was validated with the inhibition of all disinfectants tested. The total number of viable cells was determined by plating on Luria Bertani agar (LBA (per litre); 10 g tryptone (Oxoid); 5 g yeast extract (Oxoid) and 15 g agar (Oxoid)) and incubation for 2 days at 30°C. The efficacy of each disinfectant was calculated as the difference between the log transformed number of living bacteria exposed to deionized water (control) and disinfectant. All bactericidal tests were performed three times on different days and with freshly prepared solutions. All strains were tested separately.

**Table 2:** Disinfectants used in this study

Disinfectant	User concentration* (%)	Active component	Content** (%)	Reference
Ethanol	70	Alcohol	100	Kemetyl Norge AS, Norway
P3 AlcoDes	undiluted	Alcohol	70	EcoLab AS, Oslo, Norway
Virkon S	1	Peroxygen Persulfate	> 30	Lilleborg AS, Oslo, Norway

* User-concentration recommended by manufacturers

** Content (%) according to label on undiluted product

### Biofilm experiment

The biofilm system setup used for this study was performed using a Drip Flow Biofilm Reactor (Biosurface Technologies Corp., Bozeman, MT; [26]), with some modifications. Overnight cultures of GFP-tagged *S*. Agona 71.3 were mixed (1:1) with overnight cultures composed of *Staphylococcus piscifermentans*, *Pantoea agglomerans *or *Pseudomonas *sp. isolates. Cells in mixed suspensions were washed twice in sterile saline water (0.85% NaCl) after centrifugation at 13000 rpm for 4 min, and then diluted tenfold in physiological saline water, of which 10 mL was aseptically introduced to the sterile drip flow chambers, each containing a clean sterile cover glass slide. Cell mixtures were allowed to adhere to the cover glass slides for two hours at ambient temperature (25°C), after which the chambers were drained and the reactor inclined at a 10° angle. The flow of medium (0.5 mL/min, 1/10-strength TSB) was then initiated by attaching the influent tubing and starting the Watson-Marlow 205 U peristaltic pump (Watson-Marlow Ld., Falmouth, England). Biofilms were grown at 25°C for 48 h, after which the drip flow reactor was positioned horizontally and the medium flow to the reactor stopped. Mono- and dual-species biofilm experiments were performed in three independent assays.

### Laser-scanning confocal microscopy (LSCM)

Horizontal plane images of the biofilms were acquired using a Leica SP5 AOBS laser scanning confocal microscope (Leica Microsystems, Norway). Biofilms were stained with red fluorescent nucleic acid strain using SYTO 61 dye (200 μL, 1 μM, Molecular probes, Invitrogen). Cover slides with stained biofilms were immediately placed in petri dishes containing a wet paper cloth saturated with deionized water to avoid dehydration. Petri dishes were then covered with aluminum foil and the dye was left to react with nucleic acids for 30 min in the dark. LSCM allowed simultaneous 3 D monitoring of GFP and SYTO 61. The excitation wavelength used for GFP was 488 nm, and the emitted fluorescence was collected at 500-600 nm. The SYTO 61 was excited at 633 nm, and the emitted fluorescence was collected at 650-700 nm. Images were collected through a 63× Leica oil immersion objective (numeric aperture, 1.4) with a z-step of 1 μm. The quantification (biovolume, μm^3^) of GFP-tagged *S*. Agona 71.3 cells in mono- and mixed-species biofilms was estimated using PHLIP [27], a Matlab-based image analysis program http://sourceforge.net/projects/phlip/.

### Statistical analysis

Statistical significance of differences in disinfection efficacy between *Salmonella *strains was tested using ANOVA in MINITAB v15.1 (Minitab Inc., State College, PA, USA). The statistical significance of differences in disinfection efficacy between disinfectants (AlkoDes, 70% ethanol and Virkon) and species (bacteria from feed factories and five *Salmonella *strains) was tested using Fishers exact test (available at http://www.langsrud.com/fisher.htm). Efficient disinfection was defined as tests resulting in > 4 log reduction, which is the required disinfection effect to pass the European surface test. Statistical significance of differences in survival between Gram positive, Gram negative and *Salmonella *strains was tested for data at 28 days exposure using ANOVA in Minitab v15.1. Statistically significant differences in biovolume quantities (μm^3^) of *S*. Agona 71.3 in mono- and mixed-species biofilms were analyzed with Tukey's test for pair wise comparisons (Minitab). All tests were performed at 5% significance level.

## Results

### Bacterial flora in Norwegian feed industry plants

A high bacterial diversity was obtained at pre-heat treatment sites compared to post-heat treatment sites in both feed Plants A and B (Table 3). In Plant A (fish feed plant), *Staphylococcus *spp. and *Bacillus *spp. were the dominant genera at sampling points before heat treatment, representing 39% and 18%, respectively, of the total isolated flora. In post-heat treatment sites, *Staphylococcus *was more dominant, comprising 74% of the total isolated strains. Some endospore-forming isolates such as *Bacillus *and *Paenibacillus *were also identified in pre-heat treatment sites of Plant A. Among the isolates identified in post-heat treatment zones, three bacterial genera were not found in pre-heat treatment zones, such as *Corynebacterium *- an innocuous environmental bacterium, *Pediococcus *- a lactic acid bacterium and *Kytococcus*, formerly belonging to the *Micrococcus *genus.

**Table 3:** Composition of the microbiota at pre- and post-heat treatment sample sites in two feed industry plants (Plant A (fish feed) and Plant B (animal feed)).

Bacterial genus	Pre-heat treatment		Post-heat treatment	
	
	No. strains	%	No. strains	%
Plant A				
*Staphylococcus*	30	39	43	74
*Bacillus*	14	18	3	5
*Curtobacterium*	3	4	-	-
*Corynebacterium*	-	-	4	7
*Paenibacillus*	-	-	3	5
*Pediococcus*	-	-	3	5
*Pantoea*	7	9	-	-
*Psychrobacter*	4	5	-	-
Other genera (*n *= 15)	19	25	2	3
Total	**77**		**58**	
				
Plant B				
*Bacillus*	2	4	19	31
*Curtobacterium*	8	14	2	3
*Paenibacillus*	1	2	24	39
*Plantibacter*	4	7	-	-
*Staphylococcus*	6	11	2	3
*Pantoea*	19	33	7	11
*Pseudomonas*	6	11	1	2
Other genera (*n *= 13)	11	19	6	10
Total	**57**		**61**	

In Plant B (animal feed plant), samples from pre-heat treatment sites were dominated by *Pantoea *(33%), *Curtobacterium (*14%), *Staphylococcus (*11%), and *Pseudomonas (11%) *while the spore-forming *Paenibacillus *and *Bacillus *dominated at post-heat treatment sites, representing 39% and 31% of the total isolated strains. Similar to Plant A, samples isolated from sites after heat treatment were dominated by spore-forming isolates such as *Paenibacillus *(37%) and *Bacillus *(29%). Strains belonging to the genera most frequently observed at sites before heat treatment, including *Pantoea, Staphylococci *and *Pseudomonas*, were also present at post-heat treatment sites, but at a lower frequency (Table 3).

### Survival abilities of resident flora and *Salmonella *at different environmental conditions

The strains used in this study were divided into three groups, Gram positive strains, Gram negative strains, and *Salmonella *strains. Gram positive isolates included *Staphylococcus *(*n *= 3), *Corynebacterium *(*n *= 2), Gram negative isolates consisted of *Pantoea *(*n *= 2) and *Pseudomonas *(*n *= 2) while *Salmonella *included isolates of *S*. Agona (*n *= 2) and one isolate each of serovars Montevideo, Senftenberg and Typhimurium.

**Figure 1:**
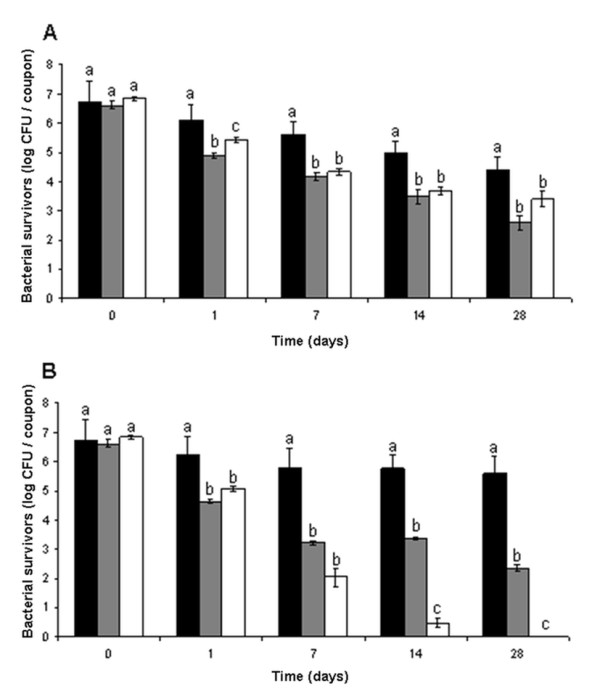
**Bacterial survival of bacteria associated with feed industry at conditions A) 35% RH and 30°C; B) 85% RH and 12°C**. Gram positives included *Staphylococcus *(*n *= 3), *Corynebacterium *(*n *= 2), Gram negatives included *Pantoea *(*n *= 2) and *Pseudomonas *(*n *= 2) while *Salmonella *included isolates of S. Agona (*n *= 2) and one isolate each of serovars Montevideo, Senftenberg and Typhimurium. Dark bars represent total tested Gram positive strains, gray bars depict Gram negatives, and white bars all tested *Salmonella *strains. Mean values of triplicate experiments are shown with error bars representing standard error of the mean. Within a group with the same incubation period (0 to 28 days; *x-*axis), mean values sharing at least one common letter are not significantly different at a *P *value of < 0.05.

Bacterial survival of *Salmonella *and dominant Gram positive and Gram negative flora isolates on stainless steel were investigated under warm and dry conditions (30°C, 35% RH) (Figure 1A), and cold and humid conditions (12°C, 85% RH) for a period of 28 days (Figure 1B). Compared to resident Gram negative bacteria, resident flora Gram positive bacteria, *Corynebacterium*, and *Staphylococcus *strains expressed higher tolerance in dry (*p *< 0.001) and humid conditions (*p*= 0.005). Gram positive bacteria also showed better survival than *Salmonella *strains under both dry (*p *< 0.001) and humid (*p*= 0.042) conditions. Under dry conditions, no significant differences in survival were observed on days 7, 14 and 28 between *Salmonella *and other Gram negative strains (*p*= 0.157). On days 14 and 28, Gram negative bacteria were better survivors in humid conditions, compared to *Salmonella *strains (p < 0.001). *Salmonella *survival was better (*p *< 0.001) at tempered and dry conditions (30°C, 35% RH) compared to cold and humid conditions (12°C, 85% RH).

### Comparison of susceptibility to disinfectants between feed factory resident bacteria and *Salmonella*

The efficiency of P3-Alcodes, 70% ethanol and Virkon S was tested in triplicate against five *Salmonella *strains originally isolated from feed factories using the European surface test. A total of 15 disinfection tests were performed for each disinfectant. For P3-Alcodes, 70% ethanol and Virkon, > 4 log reduction was obtained in 12, 10 and 10 of the disinfection tests, respectively (data not shown). No significant difference in efficiency was observed between the disinfectants used against *Salmonella*, or between *Salmonella *strains.

P3-Alcodes, 70% ethanol and Virkon S were also tested against eight dominant resident flora isolates from two feed factories (Table 1). A total of 24 disinfection tests were carried out for each disinfectant. For P3-Alcodes, 70% ethanol and Virkon, > 4 log reductions were obtained in 17, 10 and 6 of the disinfection tests, respectively (data not shown). P3-Alcodes was significantly more efficient than Virkon S (*p*= 0.0017) and 70% ethanol (*p*= 0.04). No significant differences in susceptibility between the strains were observed.

When comparing disinfection results, *Salmonella *strains were more sensitive to Virkon S than isolated resident flora strains (*p*= 0.034). When the effect of disinfectants was compared for all strains (*Salmonella *+ resident flora), P3-Alcodes was significantly more efficient than Virkon S (*p*= 0.02). No significant differences in disinfectant efficiency between P3-Alcodes and 70% ethanol (*p*= 0.09), or between Virkon S and 70% ethanol (*p*= 0.33) were observed.

### Biofilms of resident flora isolates and their influence on *Salmonella *biofilm formation

**Figure 2:**
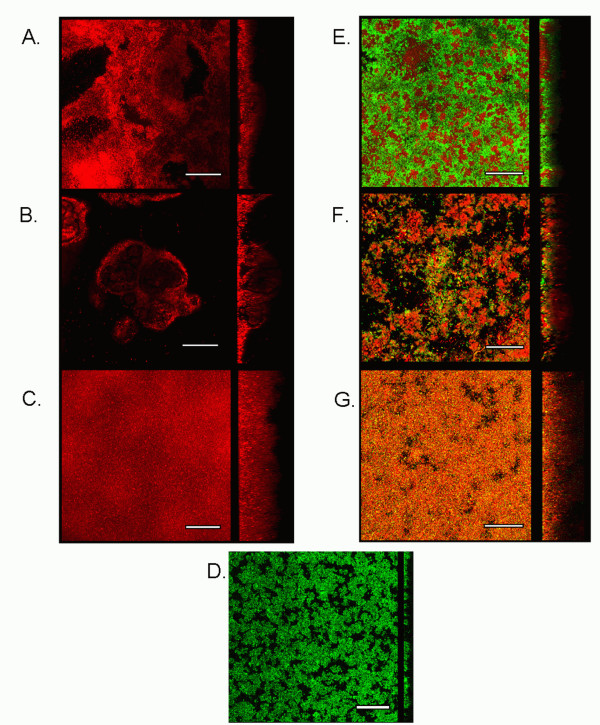
**Representative micrographs of mono- and mixed-species biofilms composed *Salmonella *Agona and selected resident flora strains**. Mono-species biofilms were composed of *Staphylococcus piscifermentans *(A), *Pantoea agglomerans *(B), *Pseudomonas *sp. (C), *S*. Agona (D), and mixed-species biofilms were composed of *S*. Agona with *S. piscifermentans *(E), *P. agglomerans *(F) and *Pseudomonas *sp. (G). Biofilms were stained using the nucleic acid dye, Syto 61, for observation using laser scanning confocal microscopy. GFP-tagged *S*. Agona cells were used. Biofilms were grown using TSB as growth medium at 25°C for two days in the Drip Flow Biofilm Reactor. For each micrograph, vertical sections of the biofilms (in the yz-plane) are presented with the left-side of each section corresponding to the substratum. In mixed-species biofilms, green cells represent pGFP-uv-tagged *S*. Agona, red cells correspond to SYTO 61-stained resident flora bacteria, and yellow cells represent GFP-tagged *S*. Agona marked with SYTO 61. Scale bars represent 50 μm.

The influence of selected *Staphylococcus piscifermentans*, *Pantoea agglomerans *and *Pseudomonas *sp. resident flora isolates on the development of *S*. Agona 71-3 cells in mixed-species biofilms, was investigated. Figure 2 illustrates representative LCSM micrographs of mono- and multi-species biofilms of resident flora and GFP-tagged *S*. Agona after two days growth using the Drip Flow Biofilm Reactor. For observation and quantification, biofilms were stained with nucleic acid dye, SYTO 61. Different biofilm architectures were observed for mono-species biofilms composed of the selected *Staphylococcus*, *Pantoea *and *Pseudomonas *strains (Figure 2ABC). *S. piscifermentans *biofilms (Figure 2A) were defined as compact, with the presence of holes in its matrix. *P. agglomerans *biofilms were highly heterogeneous, featuring "mushroom-like" super structures, while *Pseudomonas *sp. (Figure 2C) formed homogenous hyperbiofilms, characterized by compacted cells with no presence of holes in its matrix. *S*. Agona mono-species biofilms (Figure 2D) were found to be composed of more channels than resident flora mono-species biofilms. In mixed-species biofilms, *S*. Agona cells (green) were found partially covering *Staphylococcus *microcolony niches (red), while showing an increased surface coverage (*p *< 0.001) when compared to *S*. Agona cells in mono-species biofilms (Figure 2DE). In the presence of *Pantoea *(Figure 2F), *S*. Agona cells (green) were found at the bottom of the biofilm, covered by *Pantoea *cells (red), which formed super structures on top of *Salmonella *cells. In the presence of *Pseudomonas *cells (Figure 2G), both *S*. Agona (yellow) and *Pseudomonas *cells (red) were found mixed together throughout the biofilm volume (orange).

**Figure 3:**
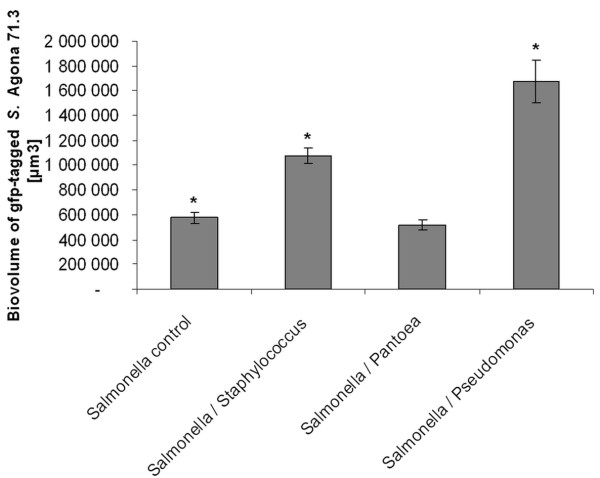
**Biovolume quantification of *Salmonella *Agona in mono- and mixed-species biofilms**. Biovolume was analyzed by PHLIP on *S*. Agona mono-species biofilms, and mixed-species biofilms composed *of S*. Agona with *Staphylococcus piscifermentans*, *Pantoea agglomerans *and *Pseudomonas *sp., after 2 days growth. Error bars indicate standard errors of mean values. Mean values sharing the symbol "*" are significantly different (*p *< 0.05).

Biofilm quantification analysis of mixed-species biofilms composed of *S*. Agona, revealed a 2.8-fold biovolume increase (*p *< 0.001) and 3.2-fold biovolume increase (*p *< 0.001) for *S*. Agona cells in mixed-species biofilms of *Staphylococcus *and *Pseudomonas*, respectively, compared to *S*. Agona mono-species biofilms (Figure 3). No significant differences in biovolume (*p*= 0.6638) were observed for *S*. Agona cells in mono-species biofilms compared to mixed-species biofilms of *Pantoea *cells.

## Discussion

While much attention is given to feed contamination by pathogenic strains in feed processing environments [6,28], there is little knowledge on the composition and survival of the environmental microbiota present throughout the feed production lines and its impact on the survival and persistence of *Salmonella*. We approached these questions by initially isolating and identifying the dominant, culturable microbiota in the two feed processing plants (one animal feed plant and one fish feed plant) followed by comparing the survival of selected resident flora strains and *Salmonella *strains to different disinfectants and environmental conditions. The impact of selected, dominant resident bacteria on the biofilm formation of *Salmonella *was also investigated.

The microbiota identified in pre-heat treatment sites from both animal and fish feed plants were mainly of environmental origins, most likely carried by incoming raw feed ingredients. Bacterial isolates belonged to genera commonly found in soil (*Staphylococcus*, *Curtobacterium*, *Rathayibacter*, *Acinetobacter*, *Sanguibacter*, *Leucobacter*), water (*Pseudomonas*, *Psychrobacter*, *Rahnella*, *Stenotrophomonas*, *Rhodococcus*), or included endospore-forming bacteria (*Bacillus*, *Paenibacillus*, *Bacillaceae*). Some of the isolated strains are known for being plant pathogens (*Erwinia, Pantoea*) or fish pathogens (*Carnobacterium*). Interestingly some common genera were isolated from both Plant A and B such as *Paenibacillus*, *Bacillus*, *Staphylococcus *and *Pseudomonas*. In Plant B, among the 10 genera identified on sites after heat treatment, six were also identified from sites located before heat treatment. This included strains within the genera *Paenibacillus*, *Bacillus*, *Pantoea*, *Staphylococcus*, *Curtobacterium *and *Pseudomonas*. For Plant A, strains within four (*Staphylococcus, Pseudomonas, Bacillus and Paenibacillus*) of the seven genera identified at sites after heat treatment were also found at sites before heat treatment.

The predominance of *Staphylococci *in post-heat treatment areas in the fish feed plant is indicative of their high tolerance to desiccation as demonstrated in previous studies [25,29]. Other studies have shown that *Staphylococci *adhere and form biofilms on a wide range of abiotic materials used in the food industry [30-32]. The lower diversity of genera observed at sites after heat treatment may indicate that certain strains have gained the ability to survive in defined feed production environments for long periods. The high presence of spore-forming strains within the genera *Paenibacillus*, *Bacillus, Aneurinibacillus, Virgibacillus *and *Brevibacillus *in post-heat treatment sites are likely to be caused by their documented heat resistance [33-37]. Although the environmental conditions in the fish feed processing plant (Plant A) could be characterized as being more humid compared to the animal feed processing plant (Plant B), the hygienic conditions of both feed plants were found to be similar. Hygienic procedures in both Plants A and B are implemented to ensure *Salmonella*-free feed products. In both plants, these include mechanical removal of soil and dust by combined scraping, brushing and vacuum cleaning, followed by disinfection at specific critical control points. Data derived from hygiene testing are also used for the assessment of required sanitary measures at specific points. Although water may be used during cleaning processes at specific points of the production process, use of water is often avoided to maintain a dry environment. In this study, Plant A reported use of water for cleaning of the extruder while no water was applied in plant B in regular cleaning processes.

No *Salmonella *strains were isolated from animal or fish feed plants in this study. However, occurrences of *Salmonella *findings in the feed industry are relatively rare. When *Salmonella *is isolated, it is usually found in pre-heat treatment sites with raw ingredients as primary origin. The same has been reported from other studies [4,9,38,39]. Since logging of RH in the production plants showed differences in RH between plant A and B, two relevant temperatures and RH conditions were selected for the surface survival studies. Although most of the *Salmonella *strains used in this study were presumed to have persisted in feed factories for years, resident flora strains survived better than all the *Salmonella *at in both humid and dry conditions over a period of 28 days. In general, survival and tolerance to desiccation were higher in Gram positive than Gram negative bacteria [40,41]. This has also been shown in other studies, for example a recent study where *Staphylococcus simulans *strains were more tolerant to desiccation compared to both *S*. Agona and *E. coli *strains [25]. Spore forming isolates were not selected in this study since the survival of spore-forming bacteria under the selected stress conditions is already well documented [42].

The uses of antimicrobial agents during cleaning and disinfection routines vary from one feed production plant to another. Although much focus is laid on the different types and specificity of antimicrobial agents currently in use by the feed industry, no study to our knowledge has investigated how resident flora isolates survive disinfection treatments. During this study, there were only small differences in disinfection efficiency against the different *Salmonella *strains, where ethanol based disinfectants and Virkon S were found to be effective against tested *Salmonella *strains dried on stainless steel. There was also a tendency for these disinfectants to be more active against *Salmonella *than the dominating non spore-forming microbiota found in both fish feed and animal feed factories. Consequently, these results suggest that the use of ethanol based disinfectants or Virkon S may reduce the *Salmonella *populations, while leaving the majority of the other bacterial populations less affected. In this study, Plant A and Plant B applied different although commonly used feed industry disinfectants. Our observations showed variable efficacy of disinfectants on *Salmonella *and the resident bacterial flora (this study; [13]) Consequently the efficacy of commonly used disinfectants on the resident microflora is relevant, as the composition of the persistent environmental bacterial flora could promote survival and biofilm formation of *Salmonella *and other potential pathogens. The effect of various disinfectants on the resident bacterial flora in feed industry premises should be further addressed in future studies.

*Salmonella *is able to persist in feed factory environments for years [6]. One recent study showed that the persistence of *Salmonella *in feed environments was correlated to its ability to form biofilms [14]. Taking into account the corroborating survival and disinfection data obtained in this and previous studies [2,13], bacterial biofilm formation is a natural and most probably an important strategy of ensuring bacterial survival. Furthermore, the formation of condensation droplets resulting from temperature variations found in some areas (post-heat treatment) of feed processing lines, may enhance biofilm formation as previously suggested [13]. Indeed, the occurrence of such biofilm niches would most likely lead to microbial colonization of new areas of the production line [43,44] and in worst cases, may become reservoirs for unwanted settled pathogenic bacteria [18]. Recent studies have reported that certain resident flora from food processing premises were able to either increase or decrease biofilm formation of *Listeria monocytogenes *[45,46] or *E. coli *[18,19]. However, it is not known whether the presence of a background flora in feed processing environments is beneficial or adverse to ensuing *Salmonella *colonization.

By using a biofilm growth model well suited for simulating conditions found in condensation environments, dominating non spore-forming resident flora isolates were found to be able to produce mature biofilms. Furthermore, beneficial effects were observed on the growth development of *Salmonella *in multispecies biofilms composed of either *Staphylococcus *or *Pseudomonas*. The presence of resident biofilms on feed processing surfaces could potentially facilitate the settlement and colonization of unwanted bacteria as demonstrated in previous studies [17,18]. Therefore, to ensure feed safety, measures should be implemented by the feed processing industry to eliminate potential biofilm forming resident flora together with specific unwanted flora.

In a preliminary experiment, potential antagonistic effects of the environmental background flora on *Salmonella *growth were also tested by agar overlay assays using BHI agar for growth of environmental bacteria overlaid with soft agar containing *Salmonella *(data not shown). Growth inhibition zones (< 3 mm diameter zones of inhibition) of *S*. Typhimurium were detected at a low frequency for only six out of approximately 4000 feed factory isolates. Thus, bacteria with significant antagonistic activity towards *Salmonella *seem not to be commonly present in feed industry environments.

It has been demonstrated in an earlier study that *Salmonella *cells could display a mutualism type of synergy in mixed community biofilms in the presence of *Klebsiella pneumoniae *[47]. Whether this type of synergism exists between feed processing *Salmonella *isolates in the presence of other resident flora isolates should be further investigated and could hold the key for better understanding the persistence properties of *Salmonella *strains in feed processing environments.

## Conclusions

This study revealed that (*i*) the survival of *Salmonella *is affected by conditions found in feed processing premises, with lower survival at a combination of low temperature and high humidity, (*ii*) resident bacterial isolates are good persisters and biofilm formers, (*iii*) commonly encountered resident bacteria as *Pseudomonas *and *Staphylococcus *may promote *Salmonella *biofilm formation in mixed-species biofilm cultivation; and (*iv*) commonly used disinfectants may differ in their bactericidal effect to various bacteria, affecting the potential biofilm formation of *Salmonella*. The presence of such mixed-species biofilms may potentially lead to colonization and persistence of *Salmonella *in feed production sites with subsequent pathogen contamination of feeds. The disinfectant efficacy of resident microflora is of relevance, as their effect may impact the biofilm formation and survival of *Salmonella *and other potential pathogens. Eliminating the establishment of resident flora isolates in feed industry surfaces is therefore of interest for impeding conditions that can lead to *Salmonella *colonization and growth on feed industry surfaces.

## Authors' contributions

OH was responsible for the study design, the performing of experiments relating to long term survival, biofilm formation of *Salmonella *and host flora strains, the analysis of data from these experiments and the preparation of the manuscript. EH was responsible for the bacterial identification of strains isolated from feed processing surfaces. TM and SL were responsible for the susceptibility study of *Salmonella *and feed factory resident bacteria to disinfectants. LKV and LLN participated in all these parts. All authors contributed to the study design and revision of the draft manuscript. All authors have read, edited and approved the final manuscript.
